# An integrated clinicopathological and genomic model for personalized prognosis in stage II-III colorectal cancer: a real-world study

**DOI:** 10.3389/fonc.2025.1742999

**Published:** 2026-01-14

**Authors:** Shuang Xie, Mingwen Kou, Zuyin Wu, Bo Sun, Jixin Zhang, Chunxu Zhang, Xianli He

**Affiliations:** 1 Department of General Surgery, Tangdu Hospital, Fourth Military Medical University, Xi’an, China; 2 Department of General Surgery, the 988th Hospital of the Joint Logistics Support Force, Zhengzhou, China

**Keywords:** clinicopathological characteristics, genomic feature, nomogram, prognosis, stage II-III colorectal cancer

## Abstract

**Background:**

The prognosis for patients with stage II-III colorectal cancer (CRC) is heterogeneous, with only a subset benefiting from adjuvant chemotherapy. Currently, prognostic models that effectively integrate clinicopathological and genetic factors remain limited. This study aimed to develop a predictive model that accurately forecasts survival and guides personalized treatment decisions.

**Methods:**

Data from 322 CRC patients were analyzed. Significant prognostic factors were selected using univariate Cox regression analysis. Subsequently, the least absolute shrinkage and selection operator (LASSO) regression algorithm, coupled with the Cox proportional hazards model, was applied to identify the most parsimonious set of predictors. A nomogram was constructed based on a multivariable Cox regression model to estimate 3- and 5-year overall survival (OS). Predictive performance was assessed using the concordance index (C-index), receiver operating characteristic (ROC) curve analysis, and calibration plots. Decision curve analysis (DCA) was performed to assess the clinical utility of the nomogram.

**Results:**

Multivariate analysis identified tumor stage, tumor differentiation grade, lymphovascular invasion, BRAF mutation, and adjuvant chemotherapy as independent predictors of OS. The developed nomogram demonstrated good discrimination, with a C-index of 0.712 and 0.726 for the training and testing cohorts, respectively. Calibration plots showed excellent agreement between predicted and observed 3- and 5-year OS. DCA confirmed that the nomogram provided clinical net benefits.

**Conclusion:**

The nomogram, integrating clinicopathological and genetic factors, provides a robust tool for predicting outcomes in patients with stage II-III CRC. It can aid in personalized treatment planning and improve patient management.

## 1 Introduction

Colorectal cancer (CRC) represents a major global health burden, with an estimated 1.9 million new cases and 930,000 deaths reported worldwide in 2020 (1). For patients with stage II-III disease, the current standard of care consists of surgical resection followed by adjuvant chemotherapy, guided by individualized risk assessment (2, 3). Despite these comprehensive therapeutic strategies, a considerable proportion of patients experience disease recurrence or progression, leading to unsatisfactory long-term outcomes. The precise identification of high-risk individuals at the time of diagnosis remains a critical unmet need in clinical oncology, as early recognition could enable timely intervention and improve prognosis (4).

In contemporary practice, prognostic stratification for stage II-III CRC, which directly informs clinical decision-making, is based on a multifactorial synthesis of patient and disease characteristics. This includes core elements such as tumor stage, clinicopathological features, surgical outcomes, and histology (5–7). The decision to administer adjuvant chemotherapy, a key component of prognosis modulation, is guided by this risk assessment, with evidence confirming a more substantial survival benefit in low-risk stage III versus high-risk stage II disease (2). In addition, the timing and duration of this chemotherapy are themselves prognostic variables (8). Finally, molecular profiling now plays an indispensable role in refining this assessment and shaping modern therapeutic strategies (9).

Advances in the understanding of CRC biology have underscored the pivotal role of molecular alterations in shaping disease behavior and therapeutic response (10). Key genetic aberrations are established determinants of critical cancer phenotypes. Specifically, mutations in driver genes such as KRAS, BRAF, and TP53 have been demonstrated to directly influence tumor aggressiveness and metastatic potential. Furthermore, these genetic alterations also govern a tumor’s sensitivity to systemic therapies (11, 12). In clinical practice, however, this information often exists in isolation from traditional staging and pathological data. For clinicians managing stage II and III CRC, this presents a challenge: while both sets of information are available, there are comparatively few practical, validated tools that synthesize them into a single, actionable prognostic profile. The scarcity of such integrated frameworks hinders the refinement of risk stratification, leaving a gap between current molecular understanding and daily clinical decision-making, ultimately preventing a more personalized approach to patient care.

To address this gap, we sought to develop and rigorously validate a comprehensive prognostic nomogram that integrates established clinical and pathological variables with genomic biomarkers. This multidimensional model aims to facilitate the early identification of high-risk patients who may benefit from intensified surveillance and individualized therapeutic strategies, thereby advancing precision oncology in CRC management.

## 2 Materials and methods

### 2.1 Patients and futures selection

A total of 322 patients with stage II-III CRC who underwent surgical treatment at Tangdu Hospital, Fourth Military Medical University, between September 2014 and September 2024 were enrolled in this study. The inclusion criteria were as follows: (a) histologically confirmed CRC; (b) elective surgical resection of the primary tumor with curative intent; and (c) pathological stage II or III disease. The exclusion criteria included: (a) receipt of any antitumor therapy before surgery (including radiotherapy, chemotherapy, or chemoradiotherapy); (b) presence of other synchronous malignancies; (c) severe hepatic disease and/or acute infection; (d) incomplete follow-up data or unknown survival outcomes; and (e) patients who died in the first postoperative month due to complications.

For each enrolled patient, demographic and clinicopathological variables were systematically documented. These included sex, age, Eastern Cooperative Oncology Group (ECOG) performance status, T stage, N stage, TNM stage, tumor location, tumor differentiation, lymphovascular invasion, and perineural invasion. Laboratory and genomic data comprised preoperative carcinoembryonic antigen (CEA) levels, microsatellite instability (MSI) status, tumor mutational burden (TMB), and mutation status in key genes (KRAS, NRAS, BRAF, TP53). Furthermore, details regarding the treatment strategies administered were also collected for analysis.

Feature selection for the prognostic nomogram was conducted in two steps. First, univariate Cox regression analyses were performed to identify variables significantly associated with OS. Variables with a *P* value < 0.05 in the univariate analysis were subsequently included in the least absolute shrinkage and selection operator (LASSO) regression model. Dummy variables were created for categorical covariates, and cross-validation was applied to determine the optimal tuning parameter (λ) for the LASSO regression. Finally, the most significant features identified by LASSO were incorporated into the final model.

### 2.2 Construction and validation of the nomogram

The most significant features identified by the LASSO regression from the training dataset were further subjected to multivariate Cox proportional hazards analysis. Variables with *P* values < 0.05 in the multivariate analysis were incorporated into the final nomogram to predict 3- and 5-year OS rates. The predictive performance of the nomogram was evaluated using time-dependent receiver operating characteristic (ROC) curves to assess its discriminative ability. Calibration plots were generated to visually compare the predicted and observed survival probabilities, thereby assessing the model’s accuracy. The clinical utility and net benefit of the nomogram were further examined using decision curve analysis (DCA).

### 2.3 Statistical analysis

All statistical analyses were performed using R software (version 4.5.1). Continuous variables were expressed as medians (range) and compared using the student’s t-test, while categorical variables were presented as counts (percentages) and compared using the chi-squared test. LASSO regression analysis was applied to the training cohort to select prognostic variables while minimizing the risk of model overfitting. Tenfold cross-validation was conducted to determine the optimal value of the tuning parameter (λ). A prognostic nomogram was constructed by integrating the six independent prognostic factors identified in the multivariate Cox analysis. Each variable was assigned a corresponding score, and the total score was calculated by summing the points of all predictors. Higher total scores were associated with shorter OS. LASSO regression analysis was conducted using the “glmnet” package. Multivariate Cox regression analyses were performed to identify independent prognostic factors, and the nomogram based on the Cox model was constructed using the “rms” package. Time-dependent ROC curves were plotted with the “timeROC” package, calibration curves were generated using the “rms” package, and DCA was performed with the “dcurves” package.

## 3 Results

### 3.1 Patient characteristics

A total of 322 patients diagnosed with stage II-III CRC were included in the final analysis. These patients were randomly divided into a training cohort (n = 225) and a validation cohort (n = 97). The overall study population comprised 145 females and 177 males. Comparative analysis of clinicopathological characteristics revealed no statistically significant differences in baseline variables between the training and validation cohorts (see **Table 1**), indicating well-balanced and comparable groups.

**Table 1:** Patient background characteristics in the training and testing cohort.

Characteristic	Training cohort (n=225)	Testing cohort (n=97)	*P*
Age, mean (SD)	62.2 (9.9)	64.3 (10.4)	0.981
Gender (%)			0.155
Female	95 (42.2)	50 (51.5)	
Male	130 (57.8)	47 (48.5)	
ECOG performance status (%)			0.565
0-1	169 (75.1)	69 (71.1)	
2	56 (24.9)	28 (28.9)	
Tumor location			0.891
Left colon or rectum	145 (64.4)	64 (66.0)	
Right colon	80 (35.6)	33 (34.0)	
T stage (%)			0.678
T1-2	81 (36.0)	38 (39.2)	
T3-4	144 (64.0)	59 (60.8)	
N stage (%)			0.734
N0	101 (44.9)	46 (47.4)	
N1	89 (39.6)	34 (35.1)	
N2	35 (15.6)	17 (17.5)	
TNM stage (%)			0.767
II	101 (44.9)	46 (47.4)	
III	124 (55.1)	51 (52.6)	
Tumor differentiation (%)			0.826
Well	58 (25.8)	27 (27.8)	
Moderate	147 (65.3)	60 (61.9)	
Poor	20 (8.9)	10 (10.3)	
Lymphovascular invasion (%)			0.241
Yes	62 (27.6)	20 (20.6)	
No	163 (72.4)	77 (79.4)	
Perineural invasion (%)			0.053
Yes	43 (19.1)	9 (9.3)	
No	182 (80.9)	88 (90.7)	
Preoperative CEA, mean (SD)	7.52 (5.58)	7.69 (6.50)	0.809
MSI Status			0.847
MSI-H	20 (8.9)	10 (10.3)	
MSS	205 (91.1)	87 (89.7)	
KRAS mutation			0.059
No	144 (64.0)	50 (51.5)	
Yes	81 (36.0)	47 (48.5)	
NRAS mutation			0.298
No	220 (97.8)	92 (94.8)	
Yes	5 (2.2)	5 (5.2)	
BRAF mutation			0.178
No	211 (93.8)	86 (88.7)	
Yes	14 (6.2)	11(11.3)	
TP 53 mutation			0.244
No	115 (51.1)	42 (43.3)	
Yes	110 (48.9)	55 (56.7)	
TMB, mean (SD)	1.72 (1.34)	2.05 (1.65)	0.071
Adjuvant chemotherapy			0.111
No	49 (21.8)	13 (13.4)	
Yes	176 (78.2)	84 (86.6)	

SD, standard deviation; ECOG, Eastern Cooperative Oncology Group; CEA, Carcinoembryonic Antigen; MSI, Microsatellite Instability; MSI-H, Microsatellite Instability-High; MSS, Microsatellite Stable; TMB, Tumor Mutational Burden.

### 3.2 Identification of independent prognostic factors for OS in the training cohort

**Figure 1:**
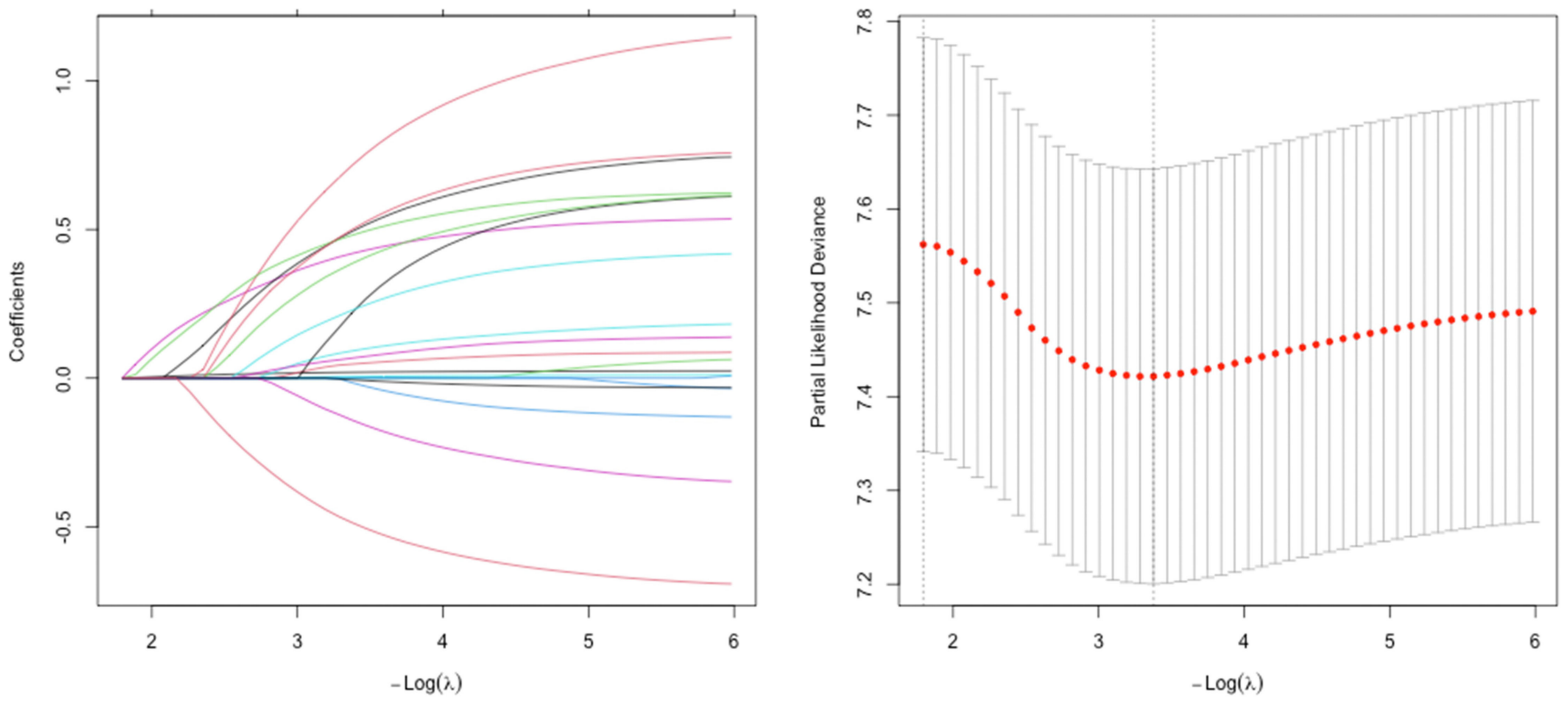
Feature selection using the least absolute shrinkage and selection operator (LASSO) regression.

The result of LASSO regression analysis was shown in **Figure 1**. The variables identified in LASSO regression were subsequently included in a multivariate Cox proportional hazards model to identify independent prognostic factors for OS. The analysis revealed that TNM stage III (HR = 3.56, 95% CI: 2.46-5.14), poor tumor differentiation (HR = 2.53, 95% CI: 1.99-3.22), presence of lymphovascular invasion (HR = 2.69, 95% CI: 1.92-3.78), BRAF mutation (HR = 5. 67, 95% CI: 3.60-8.93), absence of adjuvant chemotherapy (HR = 3.21, 95% CI: 2.23-4.61), and tumor located in the right colon (HR = 2.31, 95% CI: 1.53-3.12) were independent predictors of poor OS.

### 3.3 Construction, calibration, and validation of a nomogram

**Figure 2:**
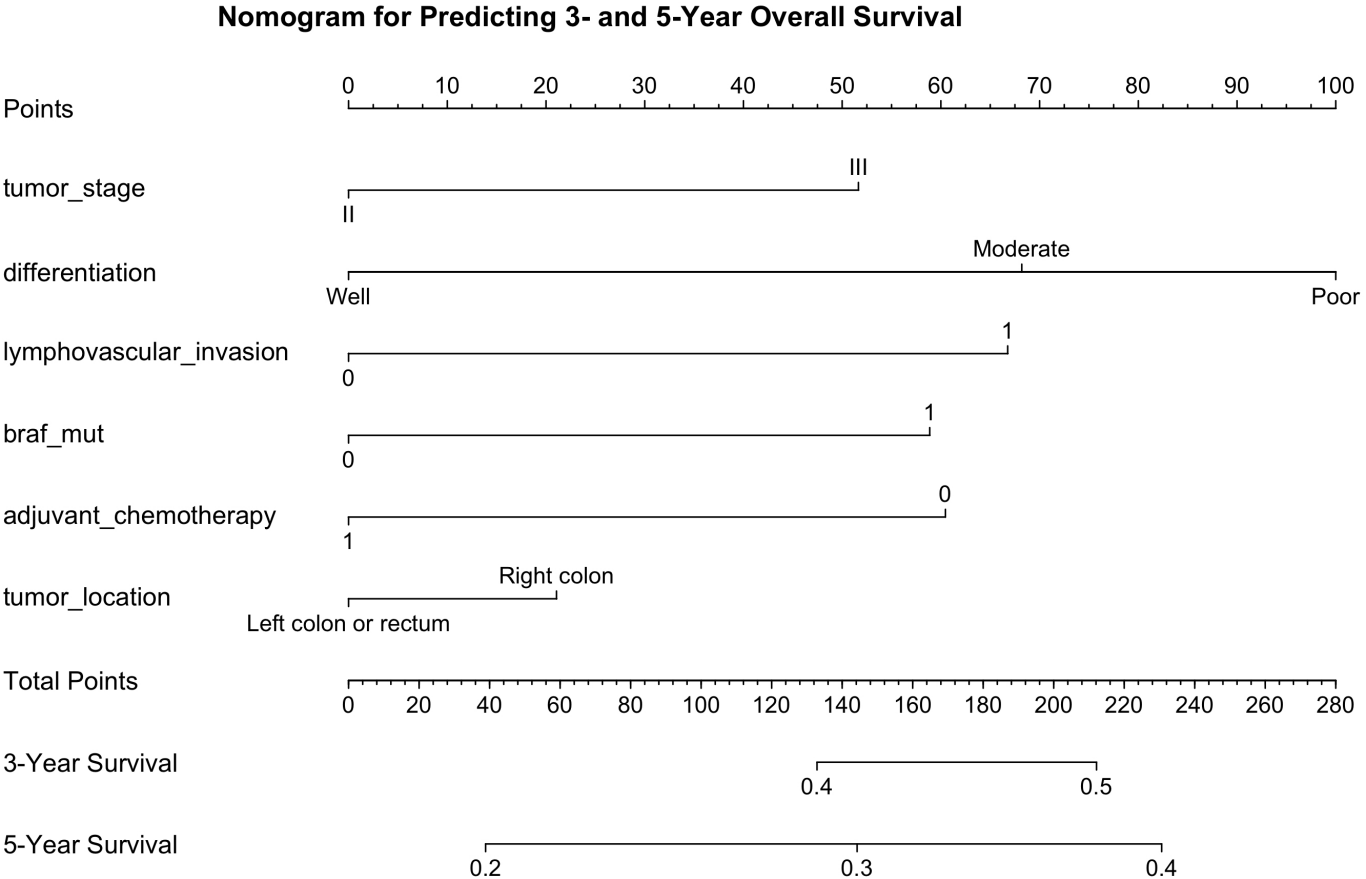
Nomogram for predicting 3- and 5-year probabilities of colorectal cancer patients (stage II-III).

**Figure 3:**
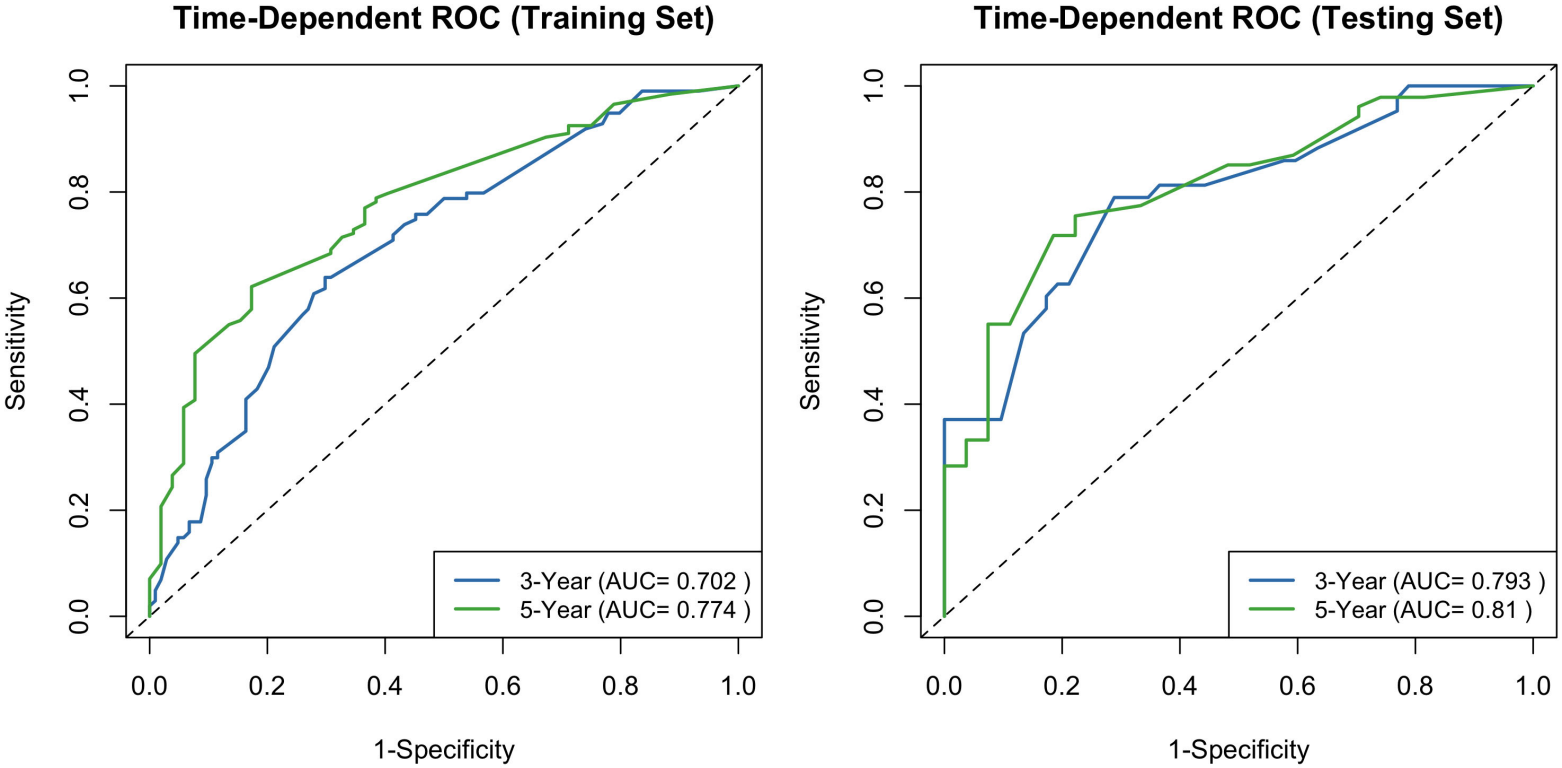
Receiver operating characteristic curves of the established nomogram for predicting 3- and 5-year OS in the training cohort and testing cohort.

**Figure 4:**
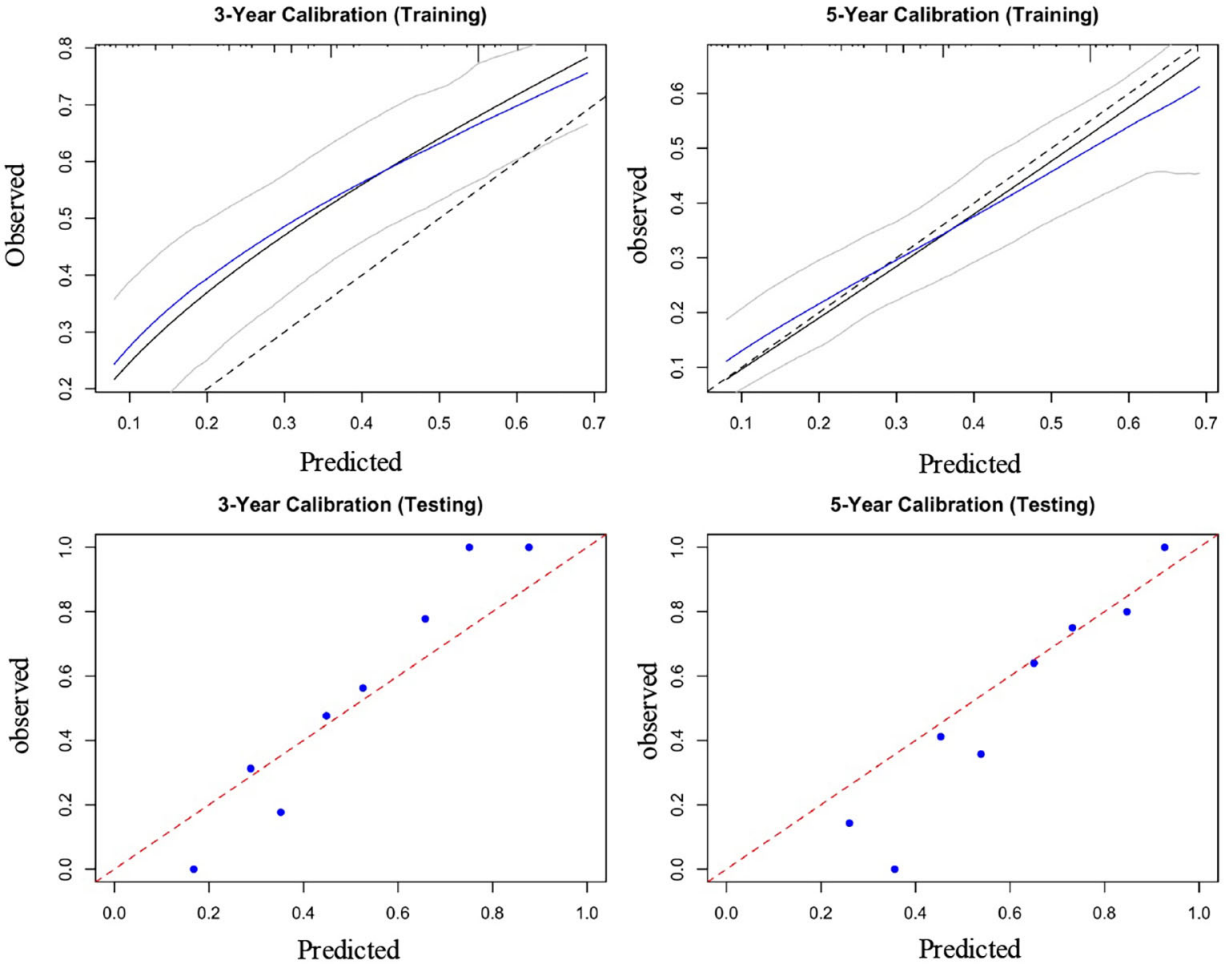
Calibration curves for survival probability in the training cohort and testing cohort.

**Figure 5:**
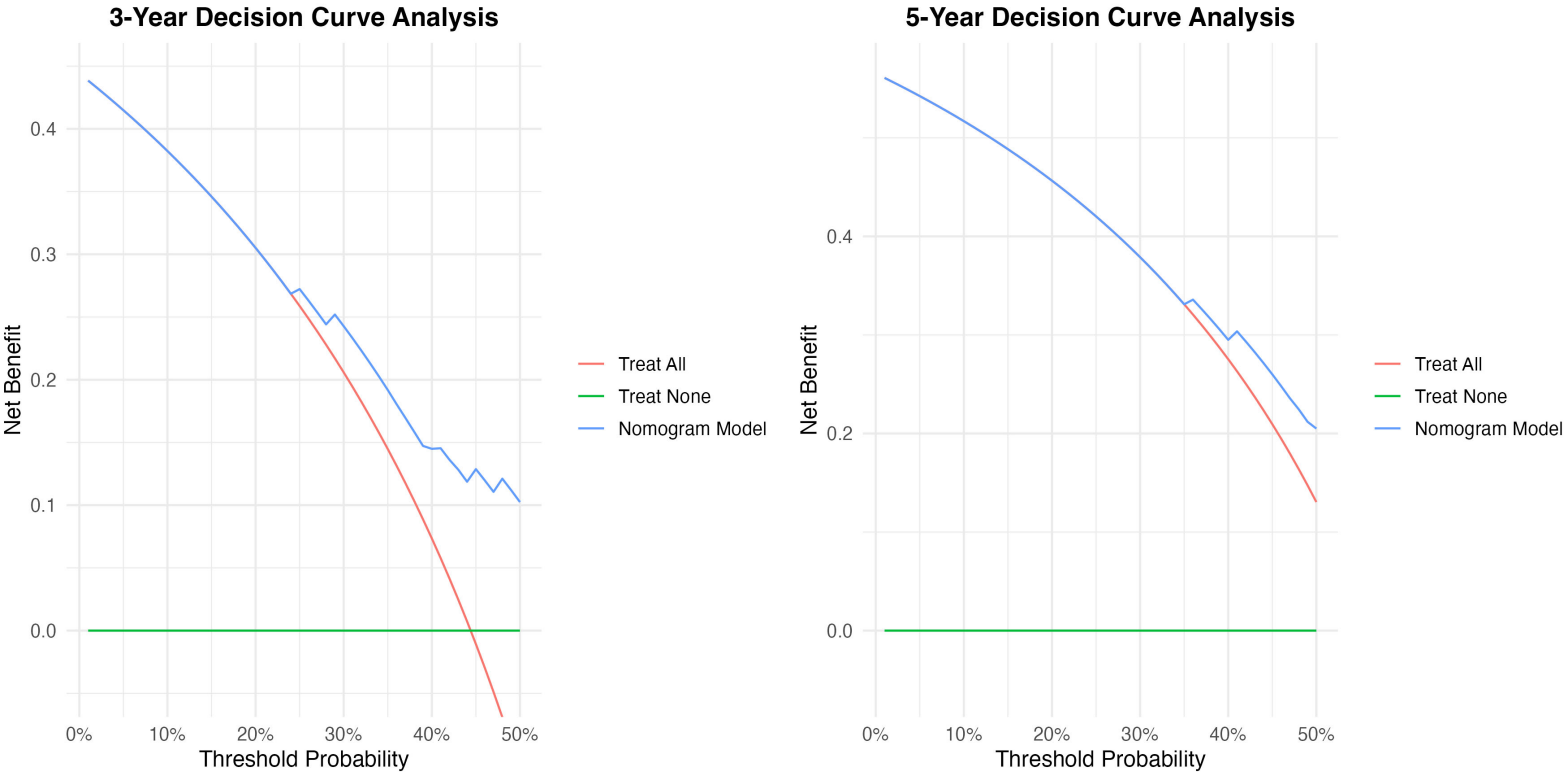
Decision curve analysis of established nomogram for 1- and 3-year OS in the training cohort and testing cohort.

By projecting a perpendicular line from the total points axis to the outcome axis, the estimated 3- and 5-year survival probabilities could be derived (as shown in **Figure 2**). The predictive performance of the nomogram was evaluated using the C-index and calibration plots. The C-index values for the training and validation cohorts were 0.712 and 0.726, respectively, indicating good discriminative ability. The areas under the ROC curves (AUCs) for predicting 3- and 5-year OS were 0.702 (95% CI: 0.601-0.791) and 0.774 (95% CI: 0.713-0.839) in the training cohort (see **Figure 3**). In the validation cohort, the corresponding AUCs for 3- and 5-year OS were 0.793 (95% CI: 0.701-0.835) and 0.810 (95% CI: 0.778-0.861, **Figure 3**). Calibration plots for both the training and validation cohorts demonstrated close agreement between predicted and observed survival probabilities (**Figure 4**), suggesting good model calibration. DCA further confirmed that the nomogram provided a meaningful net clinical benefit for predicting both 3-year and 5-year OS (**Figure 5**).

### 3.4 Risk stratification of OS

**Figure 6:**
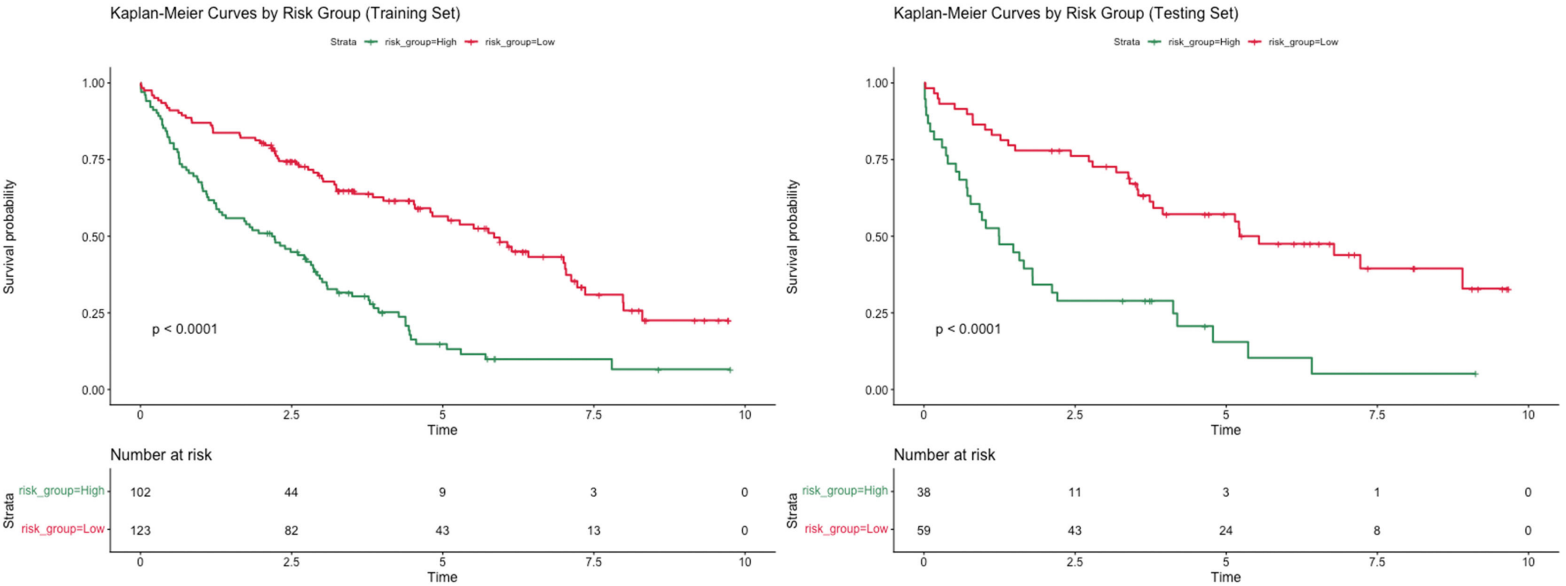
Kaplan-Meier curves for OS of high-risk patients and low-risk patients according to the optimal segmentation threshold.

A risk stratification was developed on the basis of scores calculated by the nomogram model to assess the clinical predictive ability of the nomogram model. Patients were divided into high-risk and low-risk groups based on the optimal segmentation threshold. Kaplan-Meier curves were drawn for both groups; patients in the two different risk subgroups showed significant differences in OS (P < 0.001) (**Figure 6**).

## 4 Discussion

In the present study, we identified and integrated a set of key clinical, pathological, and molecular variables to construct a robust prognostic model for stage II-III CRC. This variable set comprised tumor stage, differentiation grade, lymphovascular invasion, BRAF mutation status, receipt of adjuvant chemotherapy, and tumor location. The strength of our model lies not only in its derivation through advanced statistical techniques (LASSO and multivariate Cox regression) but also in its successful internal and external validation, confirming its accuracy in predicting 3- and 5-year OS.

Our analysis reinforces the enduring prognostic significance of key pathological factors, including advanced tumor stage, poor differentiation, and lymphovascular invasion, in patients with CRC (13–15). Furthermore, their sustained impact in our model, even after adjustment for molecular markers like BRAF, underscores their fundamental biological importance. Specifically, lymphovascular invasion, which represents a critical step in the metastatic cascade, served as a potent marker of aggressive tumor biology and heightened recurrence risk (16). This finding is strongly corroborated by our results.

A central feature of our model is the incorporation of BRAF mutation status, a well-recognized indicator of poor prognosis in CRC (17, 18). Our findings align with accumulating evidence demonstrating that the BRAF V600E mutation predicts inferior survival in stage II-III disease, particularly within MSS tumors (19, 20). Mechanistically, this adverse prognostic effect can be attributed to two key factors. First, BRAF-mutated tumors often display distinct pathological and molecular features, including serrated morphology and right-sided localization, which collectively contribute to more aggressive clinical behavior (21). Second, consistent with prior reports, BRAF-mutated MSS tumors exhibit diminished responsiveness to standard 5-fluorouracil-based adjuvant chemotherapy. The adverse prognostic influence of a BRAF mutation is notably attenuated in MSI-H tumors (22, 23). This observation underscores the complex interplay between key genomic pathways. It further validates the necessity of developing integrated prognostic models, like the one we present, that can account for such interactions.

Our results also reaffirm the pivotal role of adjuvant chemotherapy as a determinant of survival in stage II–III CRC. The ongoing clinical challenge lies in optimizing patient selection for adjuvant treatment. The current model contributes meaningfully to this effort by enabling the early identification of high-risk subgroups most likely to benefit from intensified therapy. This is particularly relevant for patients with MSS tumors harboring BRAF mutations, who generally experience poor outcomes with current standards of care. For this subset, alternative therapeutic strategies, such as irinotecan-based regimens or novel targeted and immune-based approaches, warrant further exploration.

The robust association of key pathological factors (advanced stage, poor differentiation, lymphovascular invasion) with patient outcomes in our model underscores their enduring prognostic role. While these features are cornerstones of CRC risk stratification, their persistent independence even after adjusting for the potent molecular marker BRAF underscores their fundamental and non-redundant role in tumor biology. This finding suggests that the aggressive phenotypes captured by these pathological traits are mediated through biological pathways that extend beyond, or operate independently of, the MAPK signaling cascade governed by BRAF (24–26). Particularly, lymphovascular invasion is not merely a histological observation but represents a critical milestone in the metastatic cascade, wherein tumor cells acquire the ability to intravasate into circulatory channels (27, 28). Our data strongly suggest that this event is a hallmark of a profoundly aggressive tumor phenotype, capable of driving disease progression irrespective of BRAF status. The inclusion of these robust pathological variables, in synergy with molecular data, creates a more holistic and biologically grounded model, capturing both the anatomical and molecular dimensions of tumor aggressiveness.

Despite the clinical value of our prognostic model, several limitations should be acknowledged. First, the single-center, retrospective design introduces potential selection bias and limits the generalizability of the findings. The patient cohort and treatment patterns at a single institution may not reflect broader, more heterogeneous populations. Furthermore, our cohort was restricted to patients undergoing elective surgery, excluding those requiring emergency interventions, which may further affect the model’s applicability to all-comer CRC populations. Additionally, details regarding the formal pathological documentation of complete mesocolic excision were not systematically captured in our retrospective data, which may influence surgical quality assessment and outcomes. Second, although the model demonstrated good performance in internal validation, independent external validation using multicenter cohorts is lacking and will be essential to confirm its robustness across diverse clinical settings. Third, while our genomic analysis focused on selected mutations, it did not include emerging biomarkers. Future models could be enhanced by incorporating factors like consensus molecular subtypes, mutational signatures, and the tumor immune microenvironment to improve risk stratification. Finally, the retrospective nature of follow-up may introduce inconsistencies in data collection and monitoring compared with prospective studies. Future large-scale, prospective, multicenter studies incorporating comprehensive molecular and immunological profiling and standardized surgical documentation are warranted to validate and extend our findings. Such efforts may ultimately facilitate more precise, individualized therapeutic strategies for patients with stage II-III CRC, advancing the paradigm of precision oncology in this disease.

## 5 Conclusion

In conclusion, the prognostic nomogram we have developed provides a clinically useful tool that synthesizes traditional pathological risk factors with modern molecular diagnostics. It offers a personalized and quantitative method for risk stratification, thereby aiding clinicians in optimizing postoperative management and patient counseling for stage II-III CRC. Future studies should focus on the prospective validation of this model and on exploring its utility in guiding specific therapeutic interventions.

## Data Availability

The original contributions presented in the study are included in the article/supplementary material. Further inquiries can be directed to the corresponding author.
